# Supplementary material for: Imaging correlates of visual function in multiple sclerosis
Source: PLoS One. 2020 Aug 3;15(8):e0235615. doi: 10.1371/journal.pone.0235615 (PMC7398529; doi:10.1371/journal.pone.0235615)
Supplement: S1 Table — We reported the R squared. The significance of each variable selected by PLS is reported as “Variable Importance in the Projection” (VIP). The scale coefficient, representative of the effect size for each variable, is also shown. ON = optic neuritis; MWF = myelin water fraction; GCL = Ganglion Cell Layer; OR = optic radiation; pRNFL = peripapillary retinal nerve fiber layer; GM = gray matter. (DOCX) [file pone.0235615.s001.docx]

**S1 Table.** Schematic representations of the LCLA model results looking at the entire cohort. We reported the R squared. The significance of each variable selected by PLS is reported as “Variable Importance in the Projection” (VIP). The scale coefficient, representative of the effect size for each variable, is also shown. ON = optic neuritis; MWF = myelin water fraction; GCL = Ganglion Cell Layer; OR = optic radiation; pRNFL = peripapillary retinal nerve fiber layer; GM = gray matter.

| **LCLA MODEL** | | **Variable** | **VIP** | **Scale Coefficient** |
| --- | --- | --- | --- | --- |
| **WHOLE COHORT** | **R squared 26.8** | GCL | 1.189 | 0.2723 |
|  |  | Cortical GM volume | 1.049 | 0.2402 |
|  |  | Optic radiation MWF | 0.901 | 0.2063 |
|  |  | Thalamic MWF | 0.82 | 0.1877 |
