# Supplementary material for: Imaging correlates of visual function in multiple sclerosis
Source: PLoS One. 2020 Aug 3;15(8):e0235615. doi: 10.1371/journal.pone.0235615 (PMC7398529; doi:10.1371/journal.pone.0235615)
Supplement: S2 Table — Data are mean (SD). Volumes are reported as (mm3). MD 10−3 mm2/s. (DOCX) [file pone.0235615.s002.docx]

**S2 Table.** OCT and MRI metrics values are reported for subject with negative and positive history of ON. Data are mean (SD). Volumes are reported as (mm^3^). MD 10^−3^ mm^2^/s.

|  | **ON negative history group** | **ON positive history group** |
| --- | --- | --- |
| **GCL** | 43.3(5.6) | 38.6(5.4) |
| **pRNFL** | 91.4(10.5) | 84.6(9.6) |
| **MV** | 3.0(0.1) | 3.0(0.1) |
| **VEP** | 125.7(8.6) | 129.0(11.4) |
| **Thalami MD** | 0.73(0.06) | 0.73(0.6) |
| **Thalami odi** | 0.32(0.02) | 0.33(0.02) |
| **Thalami MWF** | 56.33(12.97) | 62.37(18.47) |
| **OR MD** | 0.72(0.04) | 0.71(0.04) |
| **OR odi** | 0.59(0.05) | 0.57(0.13) |
| **OR MWF** | 0.54(0.02) | 0.51(0.11) |
| **OR lesion volume** | 1375.3(2256.5) | 1079.71 (1296.1) |
| **V1 MD** | 1.62(0.11) | 1.58(0.09) |
| **V1 odi** | 0.97(0.05) | 0.98(0.06) |
| **V1 MWF** | 121.43(35.00) | 124.20(34.35) |
| **Cerebellar MD** | 0.70(0.04) | 0.69(0.04) |
| **Cerebellar odi** | 0.46(0.03) | 0.45(0.02) |
| **Cerebellar MWF** | 51.50(13.35) | 60.40(27.71) |
| **Cortical GM volume** | 438312(48672) | 459946(40332) |
| **Thalamic volumes** | 14540(1671) | 14957(1978) |
| **V1 GM volume** | 10185(1124) | 9867(1763) |
| **Cerebellar GM volume** | 104852(11213) | 108234(9502) |
